# Association between Duration of Deep Hypothermic Circulatory Arrest and Surgical Outcome in Patients with Acute Type A Aortic Dissection: A Large Retrospective Cohort Study

**DOI:** 10.3390/jcm11030644

**Published:** 2022-01-27

**Authors:** Mona Salehi Ravesh, Christine Friedrich, Jan Schoettler, Lars Hummitzsch, Gunnar Elke, Mohamed Salem, Georg Lutter, Thomas Puehler, Jochen Cremer, Assad Haneya

**Affiliations:** 1 Department of Radiology and Neuroradiology, University Hospital Schleswig-Holstein Campus Kiel, 24105 Kiel, Germany; 2 Department of Cardiovascular Surgery, University Hospital Schleswig-Holstein Campus Kiel, 24105 Kiel, Germany; Christine.Friedrich@uksh.de (C.F.); Jan.Schoettler@uksh.de (J.S.); Mohamed.Salem@uksh.de (M.S.); Georg.Lutter@uksh.de (G.L.); Thomas.Puehler@uksh.de (T.P.); Jochen.Cremer@uksh.de (J.C.); Assad.Haneya@uksh.de (A.H.); 3 Department of Anaesthesiology and Intensive Care Medicine, University Medical Center Schleswig-Holstein, Campus Kiel, 24105 Kiel, Germany; Lars.Hummitzsch@uksh.de (L.H.); Gunnar.Elke@uksh.de (G.E.); 4 German Centre for Cardiovascular Research (DZHK), Partner Site Hamburg/Kiel/Lübeck, 20251 Hamburg, Germany

**Keywords:** deep hypothermic circulatory arrest time, acute type A aortic dissection, surgical outcome

## Abstract

(1) **Background:** Deep hypothermic circulatory arrest (DHCA) with selective antegrade cerebral perfusion (ACP) is an established cerebral protection technique for the conduction of complex surgical procedures involving the aortic arch. It is controversial whether the duration of DHCA is associated with adverse outcome in patients with acute type A aortic dissection (AAAD). Our goal was to investigate whether DHCA time was associated with surgical outcome in patients undergoing a surgical treatment of AAAD. (2) **Methods:** A total of 410 patients were divided into two groups based on the DHCA time less than 60 min and equal to or longer than 60 min. (3) **Results:** Patients with longer DHCA times were significantly younger (*p* = 0.001). Intraoperatively, complex procedures with aortic arch surgery were more common in patients with longer DHCA times (*p* < 0.001). Accordingly, cardiopulmonary bypass (*p* < 0.001), cross-clamping (*p* < 0.001) and DHCA times (*p* < 0.001) were significantly longer in this group. Postoperatively, only the duration of mechanical ventilation (*p* < 0.001) and the rate of tracheotomy were significantly higher in these patients. Thirty-day mortality was satisfactory for both groups (*p* = 0.746). (4) **Conclusions:** Our results showed that improvements in perioperative management including ACP allow for the successful performance of surgical treatment of AAAD under DHCA with a duration of even longer than 60 min.

## 1. Introduction

Cerebral complications are a predominant cause of mortality and morbidity after thoracic aortic surgery [1]. In the mid-1970s, the first experience with applying deep hypothermic circulatory arrest (DHCA) to protect the central nervous system during a complex surgical procedure for the replacement of the aortic arch was reported [2]. DHCA with adjunctive selective antegrade cerebral perfusion (SACP) supports the protection of the central nervous system during a prolonged period of circularly arrest (more than 30 min) [3]. Moreover, cerebral perfusion using antegrade and retrograde cerebral perfusion strategies during hypothermic circulatory arrest are associated with reduced death and stroke risk [4].

Despite the benefits of DHCA with SACP for vital organ support during complex cardiac surgery, some centers do not prefer its use to avoid the potential adverse impact of prolonged duration of circulatory arrest on postoperative renal function [5]. It is controversial if only the grade of hypothermia, the duration of DHCA or a combination of pre-, intra- and postoperative factors is associated with mortality in patients [6,7].

The aim of the present study was to investigate whether DHCA time is associated with the clinical outcome of patients undergoing surgical treatment of acute type A aortic dissection (AAAD).

## 2. Materials and Methods

### 2.1. Patients and Study Design

Between January 2001 and May 2019, a total of 410 consecutive patients underwent a surgical treatment of an AAAD using DHCA and ACP. Patients were divided into two groups: those with a DHCA time less than 60 min (*n* = 337; 82.2%) and those with a DHCA time equal to or longer than 60 min (*n* = 73; 17.8%).

Preoperatively, contrast enhanced computed tomography (CT) was performed to detect the exact location and extension of the dissection membrane. In a few cases, the aortic dissection was discovered incidentally during magnetic resonance imaging (MRI) or coronary angiography examination or was detected during coronary angiography in patients with iatrogenic dissections. Postoperatively, patients were examined for neurological symptoms and questioned at admission for any history of neurological events. Neurological complications were consulted directly by a neurologist and categorized according to neurological assessment, followed by head and neck CT as well as, in many cases, CT angiography for the carotid arteries to estimate the extent of stroke and brain ischemia.

The primary endpoints were 30-day mortality and postoperative neurological events. Secondary endpoints were pre- and intraoperative variables, as well as the postoperative courses such as blood loss and transfusion of blood products.

### 2.2. Operative Technique

After a standard median sternotomy, a cardiopulmonary bypass (CPB) was performed with DHCA with a nasopharyngeal temperature between 20–24 °C. Venous cannulation was performed either through the femoral vein or the right atrium. Until 2010, depending on the pathology and the extent of the dissection, the arterial cannulation was performed either through the distal ascending aorta or the femoral artery. Since 2010, the standard approach in our center is the cannulation of the left ventricle transatrial via the right upper pulmonary [8]. Retrograde blood cardioplegic solution was used for myocardial protection. Antegrade cerebral perfusion with oxygenated cold blood (18 °C) was introduced through a balloon catheter inserted bilateral in arch vessels with a pressure control of 50–60 mmHg. Once the distal anastomosis was completed, de-airing was performed by restarting retrograde perfusion through the venous cannula, followed by slow antegrade perfusion of the newly cannulated prosthesis. During rewarming, appropriate procedures for the aortic root and the aortic valve were performed. Transfusion trigger was defined as that value of hemoglobin (Hb) below 10 gm/dL. Perioperatively, neuromonitoring with near-infrared spectroscopy (NIRS) was applied. The operative technique has been described in more detail in previous papers [9,10].

### 2.3. Statistical Analysis

The statistical analysis was performed with IBM SPSS statistics (version 28.0, IBM Corp., Armonk, NY, USA). The frequency distribution of the sample data was examined for deviations from the normal distribution using the Kolmogorov–Smirnov–Lilliefors test. The mean ± standard deviation was given for normally distributed, continuous variables, while non-normally distributed continuous variables were displayed as median with associated quartiles. Categorical variables were presented as an absolute number of affected patients (*n*) and the corresponding percentage (%). The chi-square test and, if necessary, the exact Fisher test were used to compare categorial variables of the two groups examined, while the Mann–Whitney U test was applied to compare non-normally distributed continuous and ordinal variables. Survival was estimated on the right-censored data of 30-day survivors using the Kaplan–Meier method and compared for differences using the log-rank test. All *p*-values ≤ 0.05 were rated as a significant difference between the two groups. Missing values were excluded pairwise and missing data>5% are indicated in the tables.

## 3. Results

### 3.1. Demographics and Clinical Characteristics of the Study Population

Relevant demographics and preoperative data of the study participants are presented in Table 1. Patients with longer DHCA times were significantly younger (58.0 ± 12.9 vs. 63.2 ± 12.8 years, *p* = 0.001) and frequently belonged to DeBakey type I (233 vs. 69, *p* < 0.001). A lower percentage of these patients were female (23.3% vs. 37.7%, *p* = 0.019). Otherwise, there were no between-group differences concerning preoperative data.

### 3.2. Intraoperative Data

Intraoperatively, complex procedures with total aortic arch replacement were significantly more common in patients with longer DHCA times (64.4% vs. 3.6%; *p* < 0.001). Accordingly, cardiopulmonary bypass time [245 vs. 154 min; *p* < 0.001], cross-clamping time [145 vs. 83 min; *p* < 0.001] and DHCA times [88 vs. 30 min; *p* < 0.001] were significantly longer due to the complexity of the performed surgical procedures (Table 2).

Moreover, the total number of received packed red blood cells (6 vs. 3, *p* = 0.001), fresh frozen plasma (4 vs. 0, *p* = 0.002), and platelet concentrate (2 vs. 0, *p* < 0.001) was administered significantly more often in this patient group.

### 3.3. Postoperative Data and Outcomes

Also postoperatively, the total number of received packed red blood cells (6 (2; 16) vs. 3 (0; 7) unit, *p* = 0.001), fresh frozen plasma (4 (0; 12) vs. 0 (0; 6) unit, *p* = 0.002), and platelet concentrate (2 (0; 3) vs. 0 (0; 2) unit; *p* < 0.001) was significantly higher in patients with longer DHCA time (Table 3). These patients required a significantly higher rate of re-intubation (26.0% vs. 15.4%, *p* = 0.030) and tracheotomy (43.8% vs. 20.2%, *p* < 0.001) with higher duration of mechanical ventilation (134 vs. 50 min, *p* < 0.001) and duration of stay in ICU (9 (4; 19) vs. 5 (2; 10) days). Patients undergoing longer DHCA presented a non-significant higher rate of postoperative neurological deficits (31.5% vs. 21.4%, *p* = 0.06). Moreover, there was no difference between both groups concerning the postoperative complications and 30-day mortality (15.1% vs. 16.6%; *p* = 0.746).

**Figure 1:**
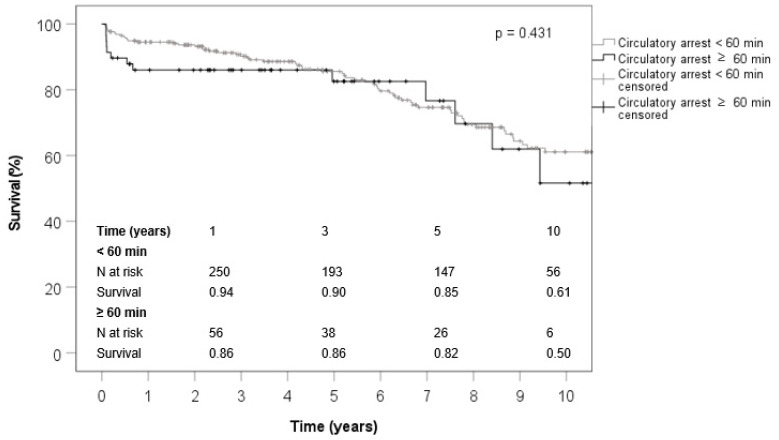
The estimated survival curves by Kaplan–Meier method.

Short- and long-term survival was satisfactory in both groups. The 1-year (94% vs. 86%), 3-year (90% vs. 86%), 5-year (85% vs. 82%) and 10-year (61% vs. 50%) survival rates were in the same range in both groups (*p* = 0.784) (Figure 1).

## 4. Discussion

In our study, the association between DHCA duration and clinical outcomes of 410 patients with AAAD was investigated. In total, 73 patients (17.8%) were identified as those with a DHCA time equal to or longer than 60 min and 337 patients (82.2%) as those with a DHCA time less than 60 min.

Both groups were compared concerning their demographic, preoperative data. However, there were significant differences between both groups concerning the intra- and postoperative data. Although the patients with longer DHCA have more complex aortic surgical procedures and correspondingly significant longer surgical duration, ICU time and postoperative in-hospital days, there was no significant difference between both groups concerning the 7-day and 30-day mortality and also long-term outcome.

In the literature, there are several studies and review articles about investigating the effect of the DHCA duration on clinical outcomes of patients during aortic surgery [3,11,12,13].

Our analysis demonstrated that patients undergoing longer DHCA presented a non-significant higher rate of postoperative neurological events (31.5% vs. 21.4%, *p* = 0.06). In a large study of a national clinical registry, O’Hara et al. [4] suggested that cerebral perfusion using antegrade and retrograde cerebral perfusion strategies are associated with reduced death and stroke risk compared with hypothermic circulatory arrest without cerebral perfusion. The impact of DHCA duration and the potential effects of ACP on mid-term quality of life (QoL) was assessed in a retrospective study of Immer et al. in 2004 [14]. In total, 363 patients with a thoracic aortic surgery were included in this study. A total of 176 patients (48.5%) of the cohort presented with an AAAD. The patients were divided into three groups according to the duration of DHCA (<20 min, 20–29 min and ≥30 min). In-hospital data comparable to our study were assessed in this study, but a much shorter follow-up (2.4 ± 1.2 years) was performed in all survivors. Immer et al. demonstrated that a DHCA duration longer than 20 min, especially longer than 35 min, adversely affected mid-term QoL in their study patients. However, the use of ACP improved the average QoL score and allowed DHCA to be extended up to 30 min, without impairment in mid-term QoL. The median DHCA is 33 min in our patients. Although we did not assess the QoL in our study, the comparison between the survivors within our patient groups during a 10-year follow-up confirm the mentioned success of DHCA with adjunctive selective ACP by Immer et al.

In a retrospective study by Mazzeffi et al. [12] in 2012, the relationship between DHCA time and perioperative bleeding and coagulopathy was investigated in a cohort of 507 consecutive patients who had thoracic aortic surgery. Mazzeffi et al. estimated the degree of bleeding and coagulopathy using perioperative transfusion. In accordance with our study, they reported a significant association between DHCA time and RBC transfusion (*p* = 0.001). In contrast to our study, they could not find any significant association between DHCA time and FFP and platelet transfusion (*p* = 0.18 and *p* = 0.06). Mazzeffi et al. reported a dependency of the association between DHCA time and the amount of bleeding (RBC transfusion) on cardiopulmonary bypass (CPB) time. They stated a positive relationship between DHCA duration and bleeding for CPB time up to 180 min but no such relationship for considerably longer CPB times (300 to 360 min). In contrast to Mazzeffi et al. our data confirm an association between longer CPB time and the amount of bleeding.

The varying evidence on deep hypothermic circulatory arrest was dealt with in a review article by Gupta et al. in 2018 [15]. In total, 17 original articles from 1999 to 2013 were reviewed in this article, which had various main foci such as neuropsychological outcome after DHCA, cerebral protection during aortic surgery, and quality of life after DHCA with ACP. Gupta et al. demonstrated that there are controversial discussions about the safe duration of DHCA. Some authors have suggested that DHCA durations in a range of 20 to 25 min affect the surgical outcomes and the quality of life [14,16], while other authors, in contrast, reported that DHCA durations under 50 min are safe for performing aortic arch interventions [17]. In a retrospective study with 656 patients, an increased incidence of postoperative stroke was reported as a result of DHCA durations of longer than 40 min [18]. In contrast, DHCA less than 60 min was demonstrated as an adequate cerebral protection technique in a comparably homogeneous and smaller study with 200 patients [19]. In a further review article, the authors associated DHCA duration of longer than 49 min with a higher rate of stroke compared to the duration of 40 to 49 min [11]. Based on a multivariable analysis in this article, DHCA times equal to and longer than 50 min significantly correlated with adverse outcomes and early death [11]. In comparison with some studies in the literature, our study included a homogenous and relatively large cohort of 410 patients with AAAD. Therefore, the surgical outcome of patients in two different DHCA times of under 60 min and equal to or above 60 min could be investigated within one study with a similar surgical setup. Our patients with longer DHCA times were reintubated significantly more frequently and underwent a common tracheotomy. They also had a significantly longer ventilation time and ICU stay time. Nevertheless, we could not observe any effect of DHCA time on cardiac and neurologic outcomes after DHCA in terms of postoperative myocardial infarction, delirium and TIA/stroke.

It is to be expected that longer DHCA time is associated with a significant higher morbidity and mortality [12,20,21]. However, the current advances in surgical techniques, circulatory management and postoperative care improve the early and late clinical outcome of patients with DHCA times of longer than 60 min [13].

The main limitations of our study were the retrospective design and the inhomogeneity of both patient groups without a propensity score matching analysis. Multivariable logistic regression analysis was performed based on this large sample size to adjust for known confounders, however, there remains a risk of unknown or not surveyed confounders.

## 5. Conclusions

Based on our 18-year single-center experience, we investigated the effect of DHCA duration on clinical outcomes in patients with AAAD. Our results confirmed that improvements in perioperative management including ACP allow for a successful performance of surgical treatment of AAAD under DHCA with a duration of even longer than 60 min. Further prospective, multicentric and randomized clinical studies with a larger group of patients are required to investigate in detail if, in consideration of improved peri- and intraoperative management, the duration of DHCA still has a strong effect on clinical outcomes in patients with AAAD.

## Figures and Tables

**Table 1:** Demographic and clinical characteristics of the study population.

Variable	Total	Cardiac Arrest <60 min	Cardiac Arrest ≥60 min	*p*-Value
**Number of surgical procedures, *n* (%)**	410 (100%)	337 (82.2%)	73 (17.8%)	----
**Age, mean** ±standard deviation **years**	62.3 ± 13.0	63.2 ± 12.8	58.0 ± 12.9	**0.001**
	63.0 (53.0; 72.3)	65 (54.5; 73.0)	57 (49.5; 66.5)	
**Female, *n* (%)**	144 (35.1%)	127 (37.7%)	17 (23.3%)	**0.019**
**EuroSCORE II**	6.0 (3.8; 13.1)	6.2 (4.0; 13.4)	5.9 (3.1; 9.9)	0.065
**Body mass index (kg/m^2^)**	26.2 (23.9; 29.0)	26.2 (23.9; 28.9)	25.8 (23.9; 29.3)	0.902
**DeBakey 1, 6.1% missing, *n* (%)**	301 (78.2%)	233 (74.0%)	68 (97.1%)	**<0.001**
**Arterial hypertension, *n* (%)**	278 (67.8%)	228 (67.7%)	50 (68.5%)	0.890
**Pulmonary hypertension, *n* (%)**	7 (1.7%)	6 (1.8%)	1 (1.4%)	1.000
**Type 2 Diabetes mellitus, *n* (%)**	20 (4.9%)	19 (5.6%)	1 (1.4%)	0.225
Insulin dependent, ***n* (%)**	6 (1.5%)	6 (1.8%)	0 (0.0%)	0.596
**Hyperlipoproteinaemia, *n* (%)**	46 (11.2%)	39 (11.6%)	7 (9.6%)	0.621
**Neurological deficits** **, *n* (%)**	77 (18.8%)	64 (19.0%)	13 (17.8%)	0.815
**Chronic renal failure/insufficiency, *n* (%)**	48 (11.7%)	39 (11.6%)	9 (12.3%)	0.855
**Renal replacement therapy, *n* (%)**	7 (1.7%)	5 (1.5%)	2 (2.7%)	0.613
**COPD, *n* (%)**	29 (7.1%)	24 (7.1%)	5 (6.8%)	0.934
**Coronary heart disease, *n* (%)**	69 (16.8%)	58 (17.2%)	11 (15.1%)	0.657
**Heart rhythm, *n* (%)**				
Atrial fibrillation, ***n* (%)**	55 (13.4%)	47 (13.9%)	8 (11.0%)	0.497
**Previous PCI, *n* (%)**	26 (6.4%)	20 (6.0%)	6 (8.2%)	0.435
**Previous cardiac surgery, *n* (%)**	35 (8.5%)	27 (8.0%)	8 (11.0%)	0.414
**Peripheral vascular disease, *n* (%)**	14 (3.4%)	12 (3.6%)	2 (2.7%)	1.000
**LVEF (%)**	60 (55;70)	60 (55;70)	66 (55;70)	0.342
Diagnostic imaging using				
Computed tomography, ***n* (%)**	359 (88.0%)	297 (88.7%)	62 (84.9%)	0.375
Coronary angiography, ***n* (%)**	128 (31.3%)	108 (32.1%)	20 (27.4%)	0.428
Magnetic resonance imaging, ***n* (%)**	6 (1.5%)	5 (1.5%)	1 (1.4%)	1.000
**Marfan syndrome, *n* (%)**	9 (2.2%)	6 (1.8%)	3 (4.1%)	0.205
**Diameter of aorta (mm)**	50 (46;60)	50 (46;60)	50 (42;60)	0.508
**Calcific aortic disease, *n* (%)**	8 (2.0%)	8 (2.4%)	0 (0.0%)	0.360
**Bicuspid aortic valve, *n* (%)**	20 (5.0%)	18 (5.5%)	2 (2.8%)	0.549
**Aortic valve vitium, *n* (%)**	159 (40.2%)	141 (43.1%)	18 (26.1%)	**0.009**
Aortic valve stenosis, ***n* (%)**	11 (2.8%)	10 (3.1%)	1 (1.4%)	0.698
Aortic valve insufficiency, ***n* (%)**	141 (35.6%)	126 (38.5%)	15 (21.7%)	**0.008**
Combined Aortic valve vitium at Aortic valve replacement, ***n* (%)**	7 (1.8%)	5 (1.5%)	2 (2.9%)	0.352
**Clinical presentation**				
Acute myocardial infarction (48 h), ***n* (%)**	14 (3.4%)	12 (3.6%)	2 (2.7%)	1.000
Cardiogenic shock, ***n* (%)**	30 (7.3%)	26 (7.7%)	4 (5.5%)	0.502
CPR, ***n* (%)**	31 (7.6%)	27 (8.0%)	4 (5.5%)	0.458
**Transfer from intensive care unit, *n* (%)**	47 (11.5%)	40 (11.9%)	7 (9.6%)	0.579
**Intubated, *n* (%)**	43 (10.5%)	37 (11.0%)	6 (8.2%)	0.481

Significant *p*-values are indicated in bold. Quantitative data are presented as median with 25th and 75th percentiles. The symbol *n* indicates the number of patients in categorical data. The European System for Cardiac Operative Risk Evaluation is abbreviated to **EuroSCORE**, chronic obstructive pulmonary disease to **COPD**, percutaneous coronary intervention to **PCI**, left ventricle to **LV**, ejection fraction to **EF**, and c-reactive protein to **CPR**.

**Table 2:** Intraoperative data.

Variable	Total	Cardiac Arrest <60 min	Cardiac Arrest ≥60 min	*p*-Value
**Duration of surgery, min**	277 (229; 340)	255 (220; 311)	358 (304; 421)	**<0.001**
**Cardiopulmonary bypass time, min**	166 (136; 210)	154 (131; 190)	245 (206; 296)	**<0.001**
**Cross-clamp time, min**	92 (71; 130)	83 (67; 109)	145 (120; 202)	**<0.001**
**Circulatory arrest, min**	33 (26; 49)	30 (24; 38)	88 (70; 129)	**<0.001**
**Number of packed red blood cells, units**	2 (0; 6)	2 (0; 5)	4 (0; 7.5)	**0.019**
**Number of fresh frozen plasma, units**	0 (0; 6)	0 (0; 4)	4 (0; 6)	**0.023**
**Number of platelet concentrate, units**	2 (1; 2)	2 (1; 2)	2 (1; 2)	**0.005**
**Surgical procedure**				
Supracoronary replacement ***n* (%)**	194 (47.3%)	187 (55.5%)	7 (9.6%)	**<0.001**
Partial arch replacement ***n* (%)**	79 (23.7%)	79 (23.5%)	18 (24.7%)	0.835
Total arch replacement ***n* (%)**	59 (14.4%)	12 (3.6%)	47 (64.4%)	**<0.001**
Conduit/Bentall operation ***n* (%)**	82 (20.0%)	64 (19.0%)	18 (24.7%)	0.273
David operation ***n* (%)**	22 (5.4%)	18 (5.3%)	4 (5.5%)	1.000
Elephant-trunk ***n* (%)**	9 (2.2%)	0 (0.0%)	9 (12.3%)	**<0.001**
Additional CABG ***n* (%)**	31 (7.6%)	29 (8.6%)	2 (2.7%)	0.086
Additional aortic valve replacement ***n* (%)**	77 (18.8%)	62 (18.4%)	15 (20.5%)	0.670
**Interventional procedure**				
TEVAR (EVAR), ***n* (%)**	27 (6.6%)	17 (5.0%)	10 (13.7%)	**0.015**
**Arterial cannulation**				
Femoral artery, ***n* (%)**	73 (17.8%)	67 (19.9%)	6 (8.2%)	**0.018**
Ascending aorta, ***n* (%)**	90 (22.0%)	71 (21.1%)	19 (16.0%)	0.353
Aortic arch, ***n* (%)**	11 (2.7%)	11 (3.3%)	0 (0.0%)	0.225
Subclavian artery, ***n* (%)**	1 (0.2%)	0 (0.0%)	1 (1.4%)	0.178
Apex, ***n* (%)**	5 (1.2%)	4 (1.2%)	1 (1.4%)	1.000
Pulmonary vein, ***n* (%)**	230 (56.1%)	184 (54.6%)	46 (63.0%)	0.189
**Venous cannulation**				
Right atrium, ***n* (%)**	399 (97.3%)	329 (97.6%)	70 (95.9%)	0.421
Bicaval, ***n* (%)**	4 (1.0%)	1 (0.3%)	3 (4.1%)	**0.019**
Femoral vein, ***n* (%)**	7 (1.7%)	7 (2.1%)	0 (0.0%)	0.361

Significant *p*-values are indicated in bold. Quantitative data are presented as median with 25th and 75th percentiles. The symbol *n* indicates the number of patients in categorical data. Coronary artery bypass graft surgery to **CABG**, thoracic endovascular aortic repair to **TEVAR**, millimeter to **mm**, and endovascular aortic repair to **EVAR**.

**Table 3:** Postoperative data and outcomes.

Variable	Total	Cardiac Arrest <60 min	Cardiac Arrest ≥60 min	*p*-Value
**New-onset of Hemodialysis, *n* (%)**	89 (21.8%)	70 (20.8%)	19 (26.0%)	0.330
**48 h drainage loss, mL**	900 (500; 1500)	850 (450; 1425)	1000 (650; 1825)	0.024
**Rethoracotomy, *n* (%)**	76 (18.5%)	58 (17.2%)	18 (24.7%)	0.138
**Postoperative blood transfusion, *n* (%)**	297 (74.1%)	240 (72.9%)	57 (79.2%)	0.276
**Total number of packed red blood cells, units**	4 (0; 8)	3 (0; 7)	6 (2; 16)	**0.001**
**Total number of fresh frozen plasma, units**	2 (0; 6)	0 (0; 6)	4 (0; 12)	**0.002**
**Total number of platelet concentrate, units**	0 (0; 2)	0 (0; 2)	2 (0; 3)	**<0.001**
**Postoperative status**				0.156
Stable, ***n* (%)**	92 (23.1%)	73 (22.3%)	19 (26.8%)	----
Stable with low dose catecholamines, ***n* (%)**	244 (61.3%)	206 (63.0%)	38 (53.5%)	----
Stable with high dose catecholamines, ***n* (%)**	51 (12.8%)	41 (12.5%)	10 (14.1%)	----
IABP/ECLS with catecholamines, ***n* (%)**	10 (2.5%)	7 (2.1%)	3 (4.2%)	----
IABP without catecholamines, ***n* (%)**	1 (0.3%)	0 (0.0%)	1 (1.4%)	----
**Reintubation, *n* (%)**	71 (17.3%)	52 (15.4%)	19 (26.0%)	0.030
**Tracheotomy, *n* (%)**	100 (24.4%)	68 (20.2%)	32 (43.8%)	<0.001
**Re-admission to the ICU, *n* (%)**	39 (9.5%)	32 (9.5%)	7 (9.6%)	0.986
**Re-admission postoperative days, d**	5 (2;8)	5 (2;8)	3.5 (1.3;13)	0.878
**Postoperative delirium, *n* (%)**	75 (18.3%)	57 (17.0%)	18 (24.7%)	0.124
**Postoperative myocardial infarction, *n* (%)**	6 (1.5%)	4 (1.2%)	2 (2.7%)	0.290
**TIA/Stroke (CT-proofed)** **, *n* (%)**	95 (23.2%)	72 (21.4%)	23 (31.5%)	0.063
**CPR, *n* (%)**	26 (6.3%)	22 (6.5%)	4 (5.5%)	1.000
**Bronchopulmonary infection, *n* (%)**	58 (14.1%)	41 (12.2%)	17 (23.3%)	0.013
**Bacteriaemia/sepsis, *n* (%)**	19 (4.6%)	17 (5.0%)	2 (2.7%)	0.547
**Sternal wound infection, *n* (%)**	6 (1.5%)	4 (1.2%)	2 (2.8%)	0.283
**Atrial fibrillation, *n* (%)**	44 (10.8%)	38 (11.3%)	6 (8.2%)	0.436
**Ventilation time, h**	64 (19;195)	50 (18;162)	134 (29;359)	<0.001
**ICU time, d**	6 (2;12)	5 (2;10)	9 (4;19)	<0.001
**Postoperative days, d**	10 (7;19)	10 (7;18)	12 (7;20)	0.241
**7 d Mortality, *n* (%)**	43 (10.5%)	37 (11.0%)	6 (8.2%)	0.485
**30 d Mortality, *n* (%)**	67 (16.3%)	56 (16.6%)	11 (15.1%)	0.746
**Hospital Mortality**	62 (15.1%)	51 (15.1%)	11 (15.1%)	0.288
Cardiac death, ***n* (%)**	32 (51.6%)	25 (49.0%)	7 (63.6%)	-----
Cerebral death, ***n* (%)**	6 (9.7%)	5 (9.8%)	1 (9.1%)	-----
Sepsis, ***n* (%)**	2 (3.2%)	1 (2.0%)	1 (9.1%)	-----
MOF, ***n* (%)**	22 (35.5%)	20 (39.2%)	2 (18.2%)	-----

Significant *p*-values are indicated in bold. Quantitative data are presented as median with 25th and 75th percentiles. The symbol *n* indicates the number of patients in categorical data. Intra-aortic balloon pump is abbreviated to **IABP**, extracorporeal life support to **ECLS**, days to **d**, intensive care unit to **ICU**, Hour to **h**, transient ischemic attack to **TIA**, computed tomography to **CT**, c-reactive protein to **CPR**, multiple organ failure to **MOF**.

## Data Availability

The data presented in this study are available on request from the corresponding author.
